# Retraction: Correction: Emotional regulation self-efficacy and impulsivity effects on college students' risk-taking behavior: a cross-sectional study

**DOI:** 10.3389/fpsyg.2026.2023231

**Published:** 2026-10-14

**Authors:** Frontiers Editorial Office

The journal retracts the 25 July 2025 article cited above.

This Correction article is retracted because the associated original article (10.3389/fpsyg.2025.1566618) has been retracted. The Correction is therefore void.

